# Correction: Conversion of carbon black recovered from waste tires into activated carbon *via* chemical/microwave methods for efficient removal of heavy metal ions from wastewater

**DOI:** 10.1039/d5ra90013a

**Published:** 2025-02-12

**Authors:** M. M. El-Maadawy, Amir A. Elzoghby, Ahmed M. Masoud, Alzahraa M. Eldeeb, Ahmed M. A. El Naggar, Mohamed H. Taha

**Affiliations:** a Nuclear Materials Authority PO Box 530, El Maddi Cairo Egypt amirelzoghby33@gmail.com; b Chemistry Department, Faculty of Science, Mansoura University Mansoura Egypt; c Egyptian Petroleum Research Institute (EPRI) 1 Ahmed El-Zomor St., Nasr City Cairo Egypt

## Abstract

Correction for ‘Conversion of carbon black recovered from waste tires into activated carbon *via* chemical/microwave methods for efficient removal of heavy metal ions from wastewater’ by M. M. El-Maadawy *et al.*, *RSC Adv.*, 2024, **14**, 6324–6338, https://doi.org/10.1039/D4RA00172A.

The authors regret that the name of one of the authors (Alzahraa M. Eldeeb) was shown incorrectly in the original article. The corrected author list is as shown here, the original version has also been updated.

